# Dietary Laver Intake and Risk of Incident Abdominal Obesity Among Korean Adults: An 18-Year Prospective Cohort Study

**DOI:** 10.3390/nu18172767

**Published:** 2026-08-24

**Authors:** Nayeon Do, Minji Kim, Sein Kim, Deukhoon Peter Han, Won Jang, Yangha Kim

**Affiliations:** 1 Department of Nutritional Science and Food Management, Ewha Womans University, Seoul 03760, Republic of Korea; janisdo1234@naver.com (N.D.); kbkjmmj2012@naver.com (M.K.); 2 Graduate Program in System Health Science and Engineering, Ewha Womans University, Seoul 03760, Republic of Korea; 3 Fisheries Policy Research Department, Korea Maritime Institute, Busan 49111, Republic of Korea; ksi@kmi.re.kr; 4 Glocal Strategy Research Department, Korea Maritime Institute, Busan 49111, Republic of Korea; bansock@kmi.re.kr; 5 Department of Food Science and Nutrition, Dongseo University, Busan 47011, Republic of Korea

**Keywords:** laver, fiber, porphyran, taurine, abdominal obesity, longitudinal study

## Abstract

**Background/Objectives**: Abdominal obesity is a major risk factor for metabolic disorders, including type 2 diabetes and cardiovascular disease. Laver (*Porphyra* spp.), a seaweed commonly consumed in Korea, contains dietary fiber and bioactive compounds that may help reduce abdominal fat accumulation. This study aimed to investigate the association between laver intake and the incidence of abdominal obesity in Korean adults. **Methods**: Data from 5457 adults aged 40–69 years who participated in the Korean Genome and Epidemiology Study were analyzed. Laver intake was assessed at baseline using a validated 103-item food frequency questionnaire. Abdominal obesity was defined as waist circumference of ≥90 cm in men and ≥85 cm in women. Cox proportional hazards models were used to estimate hazard ratios (HRs) and 95% confidence intervals (CIs). **Results**: During 18 years of follow-up, 1202 men (39.4%) and 1117 women (46.4%) developed abdominal obesity. A higher laver intake was significantly associated with a lower risk of abdominal obesity in both sexes. Compared with participants in the lowest category of laver intake, those in the highest category had a 28% lower risk among men (HR: 0.72; 95% CI: 0.58–0.89; *p* for trend = 0.0099) and a 33% lower risk among women (HR: 0.67; 95% CI: 0.54–0.84; *p* for trend = 0.0005). These associations remained significant after additional adjustment for overall diet quality. **Conclusions**: Higher habitual laver intake was associated with a lower risk of incident abdominal obesity in Korean adults. Further prospective and intervention studies are needed to confirm these findings.

## 1. Introduction

Obesity is a major public health concern worldwide and is associated with an increased risk of type 2 diabetes, cardiovascular disease, and several types of cancer [1,2]. Among obesity phenotypes, abdominal obesity is considered particularly important because visceral adipose tissue is more metabolically active than subcutaneous adipose tissue and contributes to chronic low-grade inflammation by secreting pro-inflammatory cytokines [3,4]. Consequently, abdominal obesity is recognized as a stronger predictor of cardiometabolic disorders than general obesity [5,6].

Diet is a major modifiable determinant of abdominal obesity. Previous studies have reported that a higher consumption of fruits, vegetables, and whole grains is associated with a lower risk of abdominal obesity, whereas a higher intake of refined grains and sugar-sweetened beverages is associated with an increased risk [7]. Seaweeds have also attracted considerable attention because they contain dietary fiber and various bioactive compounds that may influence lipid metabolism, energy homeostasis, and adiposity [8,9,10]. Seaweeds are rich in dietary fiber, minerals, vitamins, and bioactive compounds, including polysaccharides and polyphenols, which have been reported to exert anti-inflammatory, antioxidant, and lipid-lowering effects [11,12]. Consequently, seaweed consumption has received increasing attention as a potential dietary strategy for obesity prevention [13].

Laver (*Porphyra* spp.) is the most widely consumed edible seaweed in Korea [14] and is commonly consumed in dried or roasted sheets. Owing to its nutritional value and the growing popularity of seaweed-based foods, laver consumption has increased worldwide [15]. Previous cross-sectional studies conducted in Korea have reported that frequent laver intake is associated with a lower prevalence of abdominal obesity [16]. Previous epidemiological studies have suggested an inverse association between seaweed consumption and obesity-related outcomes, including abdominal obesity, metabolic syndrome, and dyslipidemia [16,17]. Experimental studies have also demonstrated that bioactive compounds derived from laver may regulate adipogenesis, lipid metabolism, and inflammatory pathways [18,19]. However, prospective evidence regarding the association between habitual laver intake and development of abdominal obesity remains limited.

Studies evaluating individual foods are susceptible to confounding by overall dietary quality because individuals with higher consumption of a specific food often exhibit healthier dietary behaviors. The Healthy Eating Index (HEI) is a validated measure of overall dietary quality and has been associated with abdominal obesity [20,21]. Therefore, adjusting for the HEI may help determine whether the association between laver intake and abdominal obesity is independent of overall dietary quality.

To the best of our knowledge, no prospective study has examined the association between habitual laver intake and the risk of abdominal obesity while considering overall dietary quality. Therefore, this study investigated the association between laver intake frequency and the risk of incident abdominal obesity during an 18-year follow-up period among Korean adults, after adjusting for the HEI.

## 2. Methods

### 2.1. Study Population

The participants were drawn from the Korean Genome and Epidemiology Study (KoGES), a large community-based prospective cohort established by the Korea Disease Control and Prevention Agency to identify genetic and environmental determinants of chronic diseases among Korean adults [22]. Briefly, 10,030 adults aged 40–69 years residing in Ansan and Ansung were enrolled between 2001 and 2002 and were followed up biennially thereafter. The participants were followed up until the ninth follow-up examination (2019–2020). Detailed information regarding the study design and procedures has been previously described [22].

**Figure 1:**
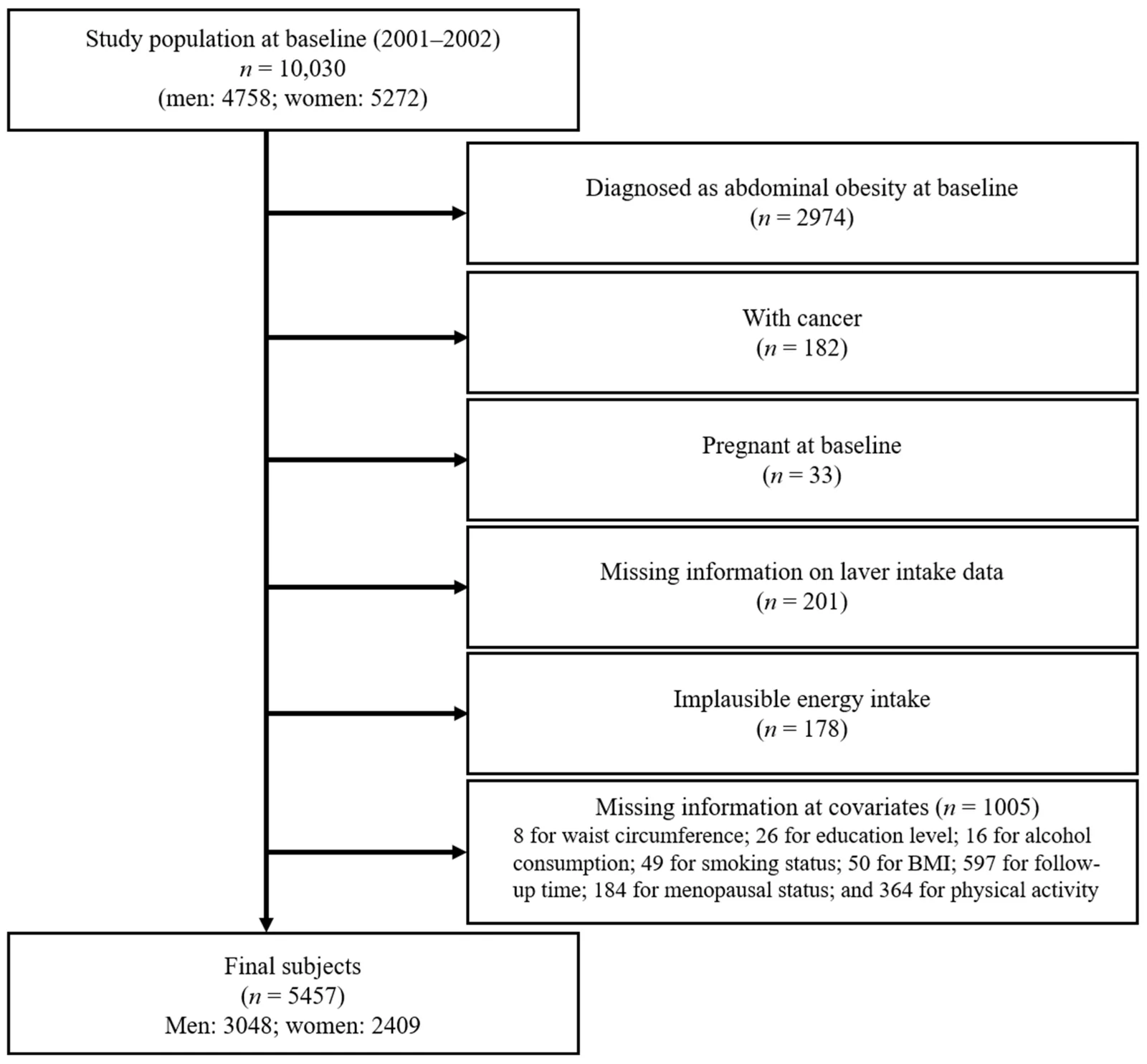
A flowchart of the study population.

Among the 10,030 participants enrolled at baseline, those with abdominal obesity (*n* = 2974) were excluded. Additional exclusion criteria included cancer or pregnancy (*n* = 215), missing laver intake data (*n* = 201), implausible total energy intake (<800 or ≥4000 kcal/day for men and <500 or ≥3500 kcal/day for women; *n* = 178), and missing covariate information (*n* = 1005). The final analytical sample comprised 5457 participants (3048 men and 2409 women) (Figure 1).

### 2.2. Ethical Approval

The study was conducted in accordance with the Declaration of Helsinki and was reviewed and determined to be exempt by the Institutional Review Board of Ewha Womans University because it involved a secondary analysis of existing de-identified data obtained from KoGES without direct interaction with the participants (IRB No. ewha-202501-0026-01; exemption approval date: 22 January 2025).

### 2.3. Assessment of Abdominal Obesity

Waist circumference (WC) was measured by trained personnel using a non-stretchable tape placed horizontally midway between the lower rib margin and the iliac crest. Measurements were obtained three times at each examination, and the average value was used for the analysis. Incident abdominal obesity was defined as a WC of ≥90 cm for men and ≥85 cm for women according to the criteria of the Korean Society for the Study of Obesity (KSSO) [23].

### 2.4. Assessment of Dietary Intake

Habitual dietary intake was assessed at baseline using a validated semi-quantitative food frequency questionnaire (SQFFQ) consisting of 103 food items developed for the KoGES [24]. Participants reported their usual consumption frequency for each food item using nine response categories ranging from “never or rarely” to “three times per day.” Nutrient intake was estimated using the Computer-Aided Nutritional Analysis Program (CAN-Pro, version 3.0; Korean Nutrition Society, Seoul, Republic of Korea) [25] together with the Korean Food Composition Table provided by the Rural Development Administration [26]. Individual food items were classified into 17 food groups, according to the KoGES protocol and a previous study (Table A1).

Laver intake was assessed as a single food item in the SQFFQ. Participants reported their usual intake frequency using nine categories: rarely or never, once per month, 2–3 times per month, 1–2 times per week, 3–4 times per week, 5–6 times per week, once per day, twice per day, or three times per day. Portion size was categorized as small (1 g), medium (2 g), or large (3 g), with 2 g defined as the standard serving size. To estimate daily laver intake, reported frequencies were converted to daily frequencies using the midpoint of each frequency category, and portion sizes were assigned factors of 0.5, 1.0, and 1.5 for small, medium, and large portions, respectively. Daily laver intake (g/day) was calculated as daily intake frequency × portion-size factor × the standard serving size (2 g). The SQFFQ assessed laver as a single food item and did not distinguish dried, roasted, seasoned, or oil-coated laver at the questionnaire level; however, both dried and seasoned laver were represented in the recipe database used for nutrient intake estimation. Participants were subsequently classified into five laver-intake frequency categories: rarely (≤1 time/month), 2–3 times/month, 1–2 times/week, 3–6 times/week, and almost every day (1–2 times/day).

Overall diet quality was evaluated using the Healthy Eating Index-2020 (HEI-2020) calculated from the SQFFQ data. The HEI-2020 consists of 13 components: nine adequacy components (total fruits, whole fruits, total vegetables, greens and beans, whole grains, dairy, total protein foods, seafood and plant proteins, and fatty acids) and four moderation components (refined grains, sodium, added sugars, and saturated fats), with a total score ranging from 0 to 100. Food and nutrient intake derived from the SQFFQ were assigned to the corresponding HEI-2020 components. Laver, a seaweed, is included in seafood and plant protein components. Higher total scores indicate greater adherence to dietary guidelines [20,27]. HEI-2020 scores were calculated according to a published scoring algorithm [20,27].

### 2.5. Assessment of Covariates

Baseline demographic, socioeconomic, lifestyle, and anthropometric characteristics were collected using standardized questionnaires and health examinations. Body mass index (BMI) was calculated as weight (kg) divided by height squared (m^2^). Physical activity was expressed as metabolic equivalent task hours per day (MET-h/day), calculated by multiplying the duration of each activity by its corresponding metabolic equivalent (MET) value and summing the values across all reported activities.

### 2.6. Statistical Analysis

Baseline characteristics are presented as means ± standard errors (SEs) for continuous variables and numbers (percentages) for categorical variables according to categories of laver intake. Differences in continuous variables across categories were evaluated using generalized linear models, whereas categorical variables were compared using chi-square tests. Tests for linear trends were performed by modeling the median value of each category as a continuous variable.

Person-years of follow-up were calculated from the baseline until the date of the first occurrence of abdominal obesity or the end of the available follow-up, whichever occurred first.

Cox proportional hazards regression models were used to estimate the hazard ratios (HRs) and 95% confidence intervals (CIs) for the incidence of abdominal obesity according to laver intake categories. For covariates that violated the proportional hazards assumption based on Schoenfeld residual tests, the interaction terms between the covariates and follow-up time were included in the models.

Covariates were selected a priori based on the previous literature and their potential association with both laver intake and abdominal obesity [16]. The multivariate model was adjusted for age (continuous), BMI (continuous), physical activity (continuous, MET-h/day), alcohol consumption (current drinker or non-current drinker), smoking status (current smoker or non-current smoker), educational level (elementary school, middle school, or high school), and total energy intake (continuous). Menopausal status was additionally included as a covariate in the models for women. The HEI-2020 score (continuous) was additionally included in the fully adjusted model.

All statistical analyses were performed using the SAS software (version 9.4; SAS Institute Inc., Cary, NC, USA). Statistical significance was defined as a two-sided *p* value < 0.05.

## 3. Results

### 3.1. Baseline Characteristics

During the 18-year follow-up period, 1202 men (39.4%) and 1117 women (46.4%) developed abdominal obesity. Baseline characteristics according to the categories of laver intake are presented in Table 1. Among men and women, participants with higher laver intake were younger and had a higher body mass index (BMI), education level, and HEI-2020 scores (all *p* < 0.05). Baseline WC showed different patterns across the laver intake categories by sex, with a decreasing trend among women but an increasing trend among men. Physical activity decreased with increasing laver intake (both *p* < 0.001), whereas the prevalence of current smoking decreased only in men (*p* < 0.05). Alcohol consumption did not differ according to laver intake in either sex.

### 3.2. Dietary Intake According to Laver Intake

Food intake according to categories of laver intake is presented in Table 2. In both men and women, higher laver intake was associated with greater consumption of legumes, nuts, fruits, vegetables, mushrooms, meat, eggs, fish, shellfish, seaweed, dairy products, and beverages (all *p* for trend < 0.01). Conversely, grain intake decreased across the laver intake categories in both sexes (both *p* for trend < 0.001). Refined grain intake also significantly decreased in men (*p* for trend < 0.001). Nutrient intakes according to categories of laver intake are shown in Table A2.

### 3.3. Cumulative Incidence of Abdominal Obesity According to Laver Intake

**Figure 2:**
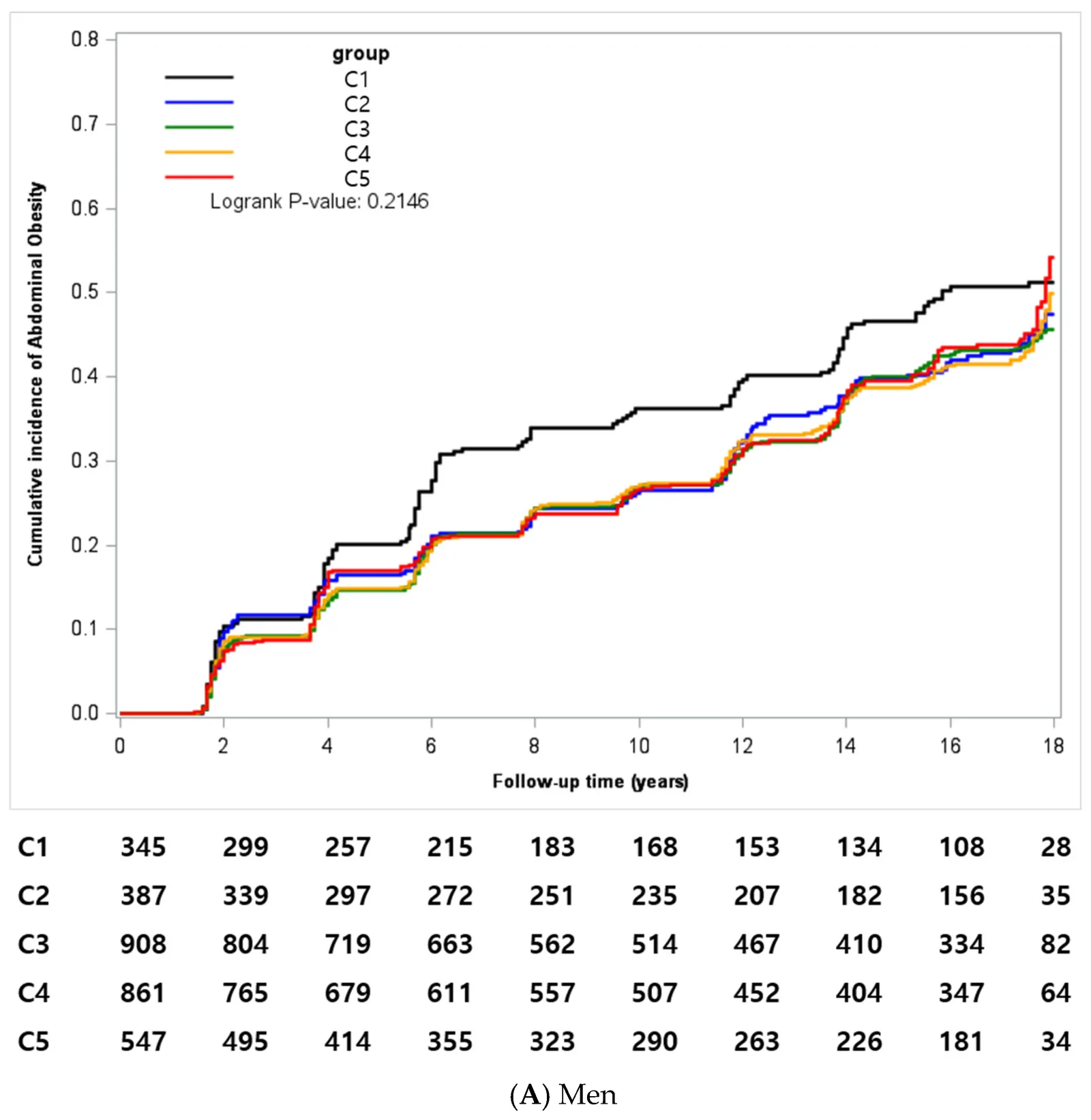

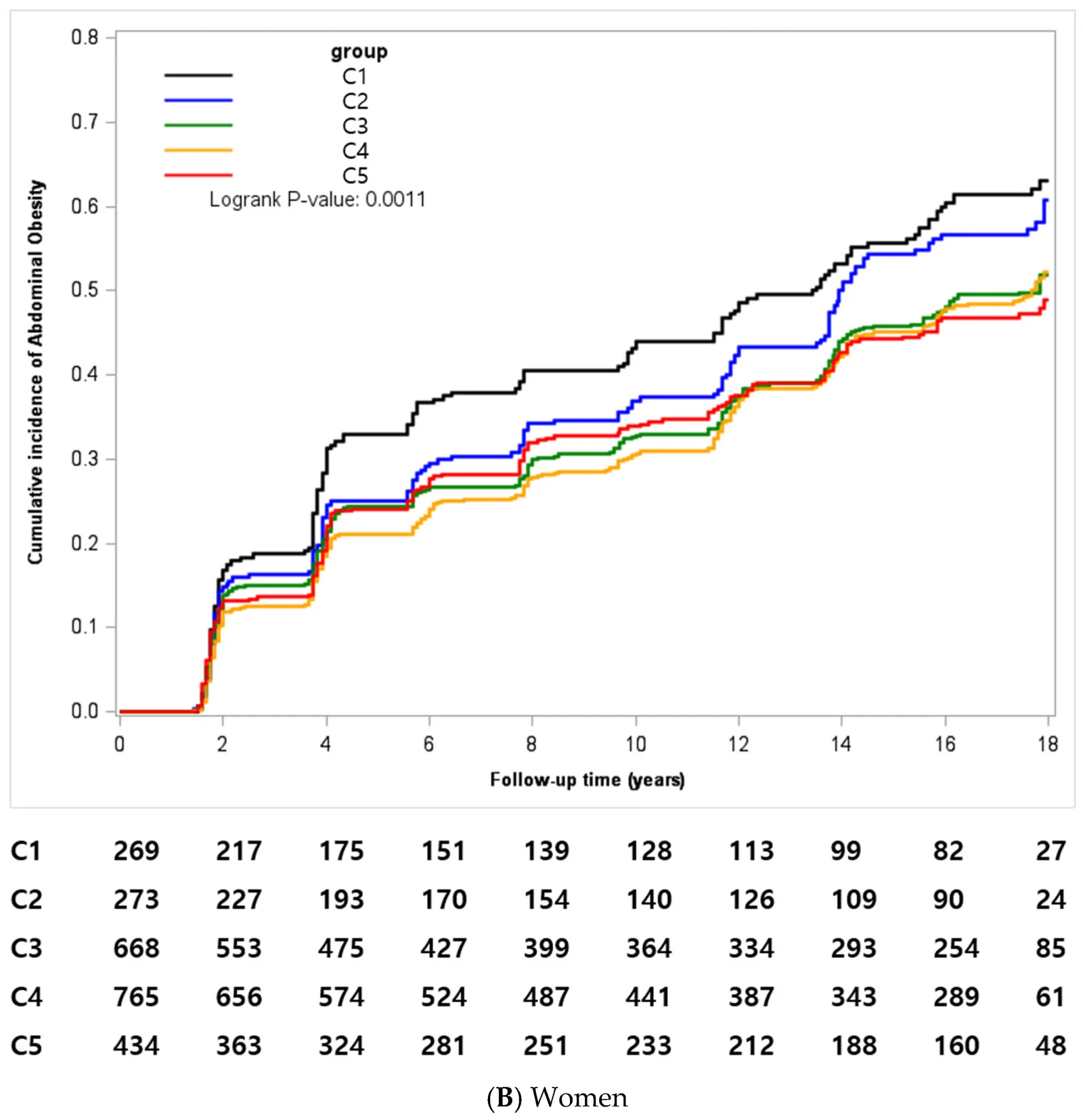
Kaplan–Meier curves for the cumulative incidence of abdominal obesity according to laver-intake frequency categories during the 18-year follow-up in (**A**) men and (**B**) women. Participants were categorized into five laver-intake frequency categories: Category 1, rarely (≤1 time/month); Category 2, 2–3 times/month; Category 3, 1–2 times/week; Category 4, 3–6 times/week; and Category 5, almost every day (1–2 times/day). Differences among the intake categories were assessed using the log-rank test.

The Kaplan–Meier curves for the cumulative incidence of abdominal obesity according to the categories of laver intake are shown in Figure 2. Among women, the cumulative incidence of abdominal obesity progressively decreased with increasing laver intake, and the differences among the categories were statistically significant (log-rank *p* = 0.0011). In contrast, no significant differences were observed among men (log-rank *p* = 0.2146).

### 3.4. Association Between Laver Intake and Incident Abdominal Obesity

The association between laver intake and the risk of abdominal obesity is presented in Table 3. In the unadjusted model (Model 1), higher laver intake was significantly associated with abdominal obesity only among women. After further adjustment for demographic and lifestyle factors (Model 2), higher laver intake was significantly associated with a lower risk of abdominal obesity in both men and women. Additional adjustments for overall diet quality using the HEI-2020 score (Model 3) produced similar results. In the fully adjusted model, men in the highest categories of laver intake had a 28% lower risk of incident abdominal obesity than those in the lowest category (HR, 0.72; 95% CI, 0.58–0.89; *p* for trend = 0.0099). Likewise, women in the highest category had a 33% lower risk of incident abdominal obesity (HR, 0.67; 95% CI, 0.54–0.84; *p* for trend = 0.0005). In a sensitivity analysis using a modified HEI-2020 score excluding the Seafood and Plant Proteins component, the inverse associations remained essentially unchanged. The HRs comparing the highest with the lowest laver-intake category were 0.71 (95% CI, 0.57–0.89; *p* for trend = 0.0084) in men and 0.67 (95% CI, 0.53–0.84; *p* for trend = 0.0005) in women.

## 4. Discussion

In this 18-year prospective cohort study, a higher laver intake was associated with a lower risk of incident abdominal obesity in both men and women. Compared with participants in the lowest category of laver intake, those in the highest category had a 28% lower risk of abdominal obesity among men and a 33% lower risk among women. Among men, the inverse association was observed only after multivariable adjustment, whereas the unadjusted model and Kaplan–Meier analysis showed no significant association. This change suggests that differences in baseline characteristics across laver-intake categories may have influenced the crude association, although the contribution of individual covariates to this shift could not be determined from the present analysis. An important finding of the present study was that these inverse associations remained statistically significant after additional adjustment for overall diet quality using the HEI-2020. As higher laver intake was accompanied by healthier food choices and substantially higher HEI-2020 scores, adjustment for overall dietary quality was important to account for the association between laver intake and healthier dietary patterns. The persistence of the association after adjusting for the HEI suggests that the observed relationship is unlikely to be explained solely by a healthier overall diet.

To the best of our knowledge, this is the first prospective cohort study to investigate the association between habitual laver intake and the incidence of abdominal obesity. Although direct prospective evidence remains unavailable, our findings are consistent with those of previous epidemiological studies. A Korean cross-sectional study involving 5777 adults reported that individuals with a higher laver intake had a lower prevalence of abdominal obesity as a component of metabolic syndrome [16]. Although the cross-sectional nature of this study precludes causal inferences, its findings are consistent with those of our longitudinal observations. In addition, a prospective dietary pattern study among middle-aged and older Korean adults demonstrated that greater adherence to a dietary pattern characterized by high consumption of seaweed, vegetables, mushrooms, fruits, tubers, soy products, and fish was associated with a lower incidence of abdominal obesity [7]. While that study evaluated overall dietary patterns rather than individual foods, seaweed was one of the principal components of protective dietary patterns, indirectly supporting our findings.

Our results are supported by experimental and systematic evidence of seaweed-derived bioactive compounds. A recent systematic review concluded that polysaccharides and other bioactive compounds isolated from edible seaweeds improved several components of metabolic syndrome, including abdominal obesity, primarily by reducing adiposity and improving lipid metabolism [28]. However, most of the previous evidence has been derived from experimental models or studies evaluating mixed seaweed consumption. Consequently, whether the habitual consumption of a single seaweed species is associated with the long-term development of abdominal obesity remains unclear. Our findings extend the existing literature by demonstrating that habitual laver intake is prospectively associated with a lower risk of abdominal obesity in a large population-based cohort.

One of the major methodological challenges in nutritional epidemiology is distinguishing the associations between individual foods and those attributable to overall dietary patterns. Individuals who frequently consume a specific food often exhibit healthier dietary behaviors in general, making confounding by the overall diet quality difficult to exclude. In the present study, participants with a higher laver intake had significantly higher educational attainment and HEI-2020 scores, suggesting that they generally adhered to healthier dietary patterns. Similar socioeconomic differences in seaweed consumption have been previously reported. A Korean study found that individuals with higher educational attainment consumed seaweed more frequently [16], whereas analyses from the Latin American Health and Nutrition Study (ELANS) demonstrated that higher socioeconomic status was associated with greater consumption of fruits, vegetables, fish, seafood, and dietary fiber [29]. These findings suggest that laver intake partly reflects an overall healthy lifestyle.

To account for potential confounders of overall dietary patterns, we adjusted for the HEI-2020 score. The HEI has been widely used to evaluate adherence to dietary guidelines and is associated with obesity-related outcomes, including abdominal obesity [20,21]. The inverse association between laver intake and abdominal obesity remained statistically significant after additional adjustment for HEI-2020, suggesting that differences in overall diet quality may not fully explain the observed association. However, the HEI-2020 was developed based on the Dietary Guidelines for Americans rather than Korean dietary guidelines. Moreover, laver contributed to the seafood and plant protein components of HEI-2020 in this study, raising the possibility of partial over-adjustment. Nevertheless, sensitivity analysis excluding the Seafood and Plant Proteins component from the HEI-2020 score yielded essentially unchanged associations, suggesting that the findings were not materially affected by this potential source of overadjustment. Therefore, the HEI-adjusted estimates should be interpreted with caution.

Several biological mechanisms may explain the inverse association between laver intake and abdominal obesity observed in this study. One plausible explanation is the high dietary fiber content of laver. Dietary fiber has consistently been associated with lower waist circumference and reduced visceral adiposity in epidemiological studies [30,31,32,33]. In our study, participants with a higher laver intake also consumed significantly more dietary fiber than those with a lower intake, suggesting that dietary fiber may have contributed, at least in part, to the observed association.

The beneficial effects of dietary fiber on abdominal obesity are likely mediated by several complementary mechanisms. Dietary fiber increases satiety by delaying gastric emptying and stimulating the secretion of appetite-regulating hormones, thereby reducing subsequent energy intake [34,35]. In addition, soluble fiber attenuates postprandial glucose and insulin responses by slowing nutrient absorption, whereas fermentation of dietary fiber by gut microbiota produces short-chain fatty acids that improve insulin sensitivity and energy metabolism [36,37,38]. Collectively, these mechanisms may contribute to reduced visceral fat accumulation and improved metabolic health.

In addition to dietary fiber, laver contains several bioactive compounds that may prevent abdominal obesity. Porphyran, a sulfated polysaccharide unique to red seaweeds, has demonstrated anti-obesity effects in experimental studies through the regulation of lipid metabolism, modulation of the gut microbiota, and activation of AMP-activated protein kinase (AMPK), which ultimately suppresses adipogenesis and promotes lipid oxidation [39,40,41,42,43,44,45]. Laver also contains taurine, which improves energy metabolism, reduces adipose tissue accumulation, and enhances thermogenesis through AMPK-dependent pathways in experimental models [46,47,48,49]. However, these mechanisms have primarily been demonstrated in experimental models, and whether habitual laver consumption provides sufficient amounts of these compounds to elicit similar effects in humans remains unclear. Thus, these findings provide biological plausibility for the observed association but should not be considered direct evidence of the mechanisms underlying the association in the present study. Further clinical and mechanistic studies are warranted to clarify the specific contributions of individual laver-derived bioactive compounds to the prevention of abdominal obesity.

Among women, participants in the highest category of laver intake had a higher baseline BMI; they had a lower waist circumference than those in the lowest category. These seemingly discrepant observations may reflect different aspects of adiposity captured by these measures. BMI reflects overall obesity and does not directly assess regional adiposity [50], whereas waist circumference provides a more accurate estimate of adipose tissue distribution [51]. Therefore, the higher BMI observed among participants with a higher laver intake may not necessarily indicate abdominal obesity.

The present study has several limitations. First, dietary intake was assessed only at baseline using the validated SQFFQ. Therefore, the changes in dietary habits during the 18-year follow-up period could not be determined. As a result, changes in habitual laver intake over time may have led to non-differential exposure misclassifications, potentially influencing the estimated associations. Some degree of measurement error and recall bias may have occurred with self-reported dietary assessments. Second, information on laver preparation methods, including seasoned versus unseasoned lavers, was not available in the present study. Therefore, we could not determine whether the higher sodium intake observed among participants with a higher laver intake was attributable to seasoned laver or other dietary sources, which may have implications for the potential cardiometabolic effects of laver consumption. Third, abdominal obesity was defined using waist circumference rather than direct measurement of visceral adipose tissue obtained using imaging techniques. Although waist circumference is a validated surrogate marker of central obesity, it cannot distinguish between visceral and subcutaneous fat. In addition, although baseline BMI was included as a measure of overall adiposity, baseline WC was not additionally included in the multivariable models; therefore, residual confounding by baseline central adiposity cannot be completely excluded. Fourth, circulating biomarkers related to laver intake, including serum taurine concentrations, were unavailable, limiting the mechanistic interpretation of the observed associations. Fifth, the HEI-2020 was developed for the U.S. population, and laver itself contributed to its seafood and plant protein components in the present study; therefore, the possibility of partial overadjustment cannot be excluded. Sixth, participants excluded because of missing covariate data differed from those included in the analysis with respect to several baseline characteristics, although laver intake did not differ significantly between the groups. Therefore, potential selection bias resulting from complete-case analysis cannot be excluded. Finally, the study population consisted of middle-aged Korean adults with a relatively high habitual seaweed consumption. Consequently, the generalizability of our findings to populations with substantially different dietary habits and cultural patterns of seaweed consumption is limited. Further studies involving diverse populations are warranted.

Despite these limitations, this study has several strengths. First, this study was based on a large population-based prospective cohort with up to 18 years of follow-up, enabling evaluation of the long-term association between habitual laver intake and incident abdominal obesity. Second, abdominal obesity was assessed repeatedly throughout the follow-up period, allowing for the identification of incident cases rather than relying on cross-sectional measurements. Third, detailed information on demographic characteristics, lifestyle factors, and dietary intake was collected using standardized protocols, enabling adjustment for a broad range of potential confounding variables.

## 5. Conclusions

In conclusion, higher habitual laver intake was associated with a lower risk of incident abdominal obesity during the 18 years of follow-up among Korean adults. Importantly, this inverse association remained statistically significant after additional adjustment for overall dietary quality using the HEI-2020, suggesting that the observed association was not solely attributable to healthier dietary patterns. However, given the observational nature of this study, these findings do not establish a causal relationship between habitual laver intake and risk of abdominal obesity. Further prospective and randomized controlled trials are warranted to confirm these findings and to elucidate the underlying biological mechanisms before dietary recommendations regarding laver intake can be made.

## Figures and Tables

**Table 1:** Baseline characteristics of study participants according to laver-intake frequency categories.

	Laver Intake Frequency Categories					
	Rarely (≤1 Time/Month)	2–3 Times/Month	1–2 Times/Week	3–6 Times/Week	Almost Every Day (1–2 Times/Day)	*p*-Value
Men						
No. of participants	345	387	908	861	547	<0.0001
Laver intake (g/day)	0.04	0.19	0.47	1.28	2.64	<0.0001
Age (years)	53.54 ± 0.48	52.64 ± 0.45	51.15 ± 0.29	50.76 ± 0.29	50.32 ± 0.37	<0.0001
BMI (kg/m^2^)	22.91 ± 0.13	22.98 ± 0.12	23.20 ± 0.08	23.61 ± 0.08	23.75 ± 0.10	<0.0001
WC (cm)	80.73 ± 0.31	80.53 ± 0.31	80.41 ± 0.19	81.31 ± 0.19	81.30 ± 0.24	0.0041
Physical activity (MET-h/d)	31.14 ± 1.04	30.73 ± 0.93	25.19 ± 0.57	24.11 ± 0.54	22.92 ± 0.63	<0.0001
Non-current drinker (%)	49 (14.20)	61 (15.76)	184 (20.26)	162 (18.82)	108 (19.74)	0.0707
Non-current smoker (%)	142 (41.16)	195 (50.39)	472 (51.98)	435 (50.52)	280 (51.19)	0.0132
Education (≥high school, %)	143 (41.45)	175 (45.22)	550 (60.57)	556 (64.58)	373 (68.19)	<0.0001
HEI-2020 score (median)	29.80	32.53	34.25	36.40	37.34	<0.0001
Women						
No. of participants	269	273	668	765	434	<0.0001
Laver intake (g/day)	0.05	0.18	0.46	1.27	2.74	<0.0001
Age (years)	53.58 ± 0.57	52.01 ± 0.55	50.75 ± 0.33	50.00 ± 0.31	50.45 ± 0.39	<0.0001
BMI (kg/m^2^)	23.12 ± 0.15	23.36 ± 0.15	23.45 ± 0.09	23.64 ± 0.09	23.67 ± 0.13	0.0210
WC (cm)	77.03 ± 0.33	76.74 ± 0.34	75.58 ± 0.22	75.78 ± 0.20	75.46 ± 0.28	0.0002
Physical activity (MET-h/d)	26.59 ± 1.09	23.50 ± 0.99	21.41 ± 0.60	21.64 ± 0.54	21.36 ± 0.73	<0.0001
Non-current drinker (%)	195 (72.49)	188 (68.86)	462 (69.16)	520 (67.97)	316 (72.81)	0.3743
Non-current smoker (%)	256 (95.17)	260 (95.24)	639 (95.66)	739 (96.60)	420 (96.77)	0.6423
Education (≥high school, %)	52 (19.33)	78 (28.57)	248 (37.13)	345 (45.10)	198 (45.62)	<0.0001
Postmenopausal women, *n* (%)	179 (66.54)	163 (59.71)	344 (51.50)	396 (51.76)	240 (55.30)	0.0012
HEI-2020 score (median)	36.92	39.47	40.53	42.34	42.76	<0.0001

Values are presented as means ± standard errors for continuous variables and numbers (percentages) for categorical variables. *p*-value was obtained from general linear models for continuous variables and the Chi-square test for categorical variables. Participants were classified into five laver-intake frequency categories: rarely (≤1 time/month), 2–3 times/month, 1–2 times/week, 3–6 times/week, and almost every day (1–2 times/day). BMI, body mass index; WC, waist circumference; MET-h/d, metabolic equivalent task-hours per day; HEI-2020, healthy eating index-2020.

**Table 2:** Food intake according to laver-intake frequency categories.

	Laver Intake Frequency Categories					
Food Intake (g/1000 kcal/Day)	Rarely (≤1 Time/Month)	2–3 Times/Month	1–2 Times/Week	3–6 Times/Week	Almost Every Day (1–2 Times/Day)	*p*-Trend
Men						
Grains	461.05 ± 4.26	446.15 ± 3.96	412.31 ± 2.36	387.91 ± 2.46	376.91 ± 2.79	<0.0001
Refined grains	606.49 ± 17.33	558.03 ± 15.68	547.83 ± 10.94	521.14 ± 11.37	493.47 ± 14.37	<0.0001
Whole grains	189.73 ± 15.30	220.84 ± 14.65	245.08 ± 10.31	270.09 ± 10.27	304.43 ± 13.15	<0.0001
Potatoes	6.30 ± 0.48	6.98 ± 0.49	7.46 ± 0.26	8.34 ± 0.32	9.06 ± 0.42	<0.0001
Sweet foods and sugars	5.07 ± 0.36	5.39 ± 0.36	6.34 ± 0.22	5.97 ± 0.19	6.66 ± 0.26	0.0022
Oil and fats	0.02 ± 0.01	0.02 ± 0.01	0.03 ± 0.01	0.03 ± 0.01	0.03 ± 0.01	0.2398
Legumes	15.03 ± 0.67	14.92 ± 0.58	16.51 ± 0.40	18.37 ± 0.42	19.09 ± 0.57	<0.0001
Nuts	0.32 ± 0.05	0.35 ± 0.08	0.38 ± 0.03	0.45 ± 0.04	0.61 ± 0.06	<0.0001
Fruits	102.84 ± 6.53	108.52 ± 6.53	103.04 ± 3.02	122.96 ± 3.76	114.60 ± 4.57	0.012
Vegetables	36.43 ± 1.67	37.35 ± 1.51	41.16 ± 0.89	46.58 ± 1.05	50.19 ± 1.57	<0.0001
Mushrooms	2.45 ± 0.26	2.53 ± 0.19	3.53 ± 0.14	4.56 ± 0.18	5.49 ± 0.29	<0.0001
Meats	24.46 ± 1.20	26.09 ± 0.98	32.06 ± 0.70	33.98 ± 0.79	33.21 ± 0.95	0.0008
Eggs	3.55 ± 0.31	4.54 ± 0.32	6.09 ± 0.22	7.38 ± 0.27	8.10 ± 0.38	<0.0001
Fish and shellfish	12.91 ± 0.70	14.48 ± 0.67	19.70 ± 0.48	24.41 ± 0.58	26.68 ± 0.77	<0.0001
Seaweed	0.19 ± 0.02	0.30 ± 0.01	0.58 ± 0.01	1.07 ± 0.02	1.70 ± 0.04	<0.0001
Dairy	36.51 ± 3.31	44.37 ± 2.91	52.05 ± 1.78	54.19 ± 1.87	55.21 ± 2.30	<0.0001
Beverages	32.29 ± 2.67	36.82 ± 2.42	45.37 ± 1.65	52.44 ± 1.70	60.99 ± 2.48	<0.0001
Women						
Grains	444.58 ± 6.19	439.27 ± 5.14	404.83 ± 3.13	375.77 ± 2.97	372.95 ± 3.61	<0.0001
Refined grains	386.85 ± 20.28	406.82 ± 19.20	378.06 ± 11.93	354.20 ± 11.01	376.87 ± 15.21	0.2759
Whole grains	315.44 ± 19.33	310.90 ± 18.28	321.94 ± 11.54	335.14 ± 10.58	362.34 ± 14.58	0.0067
Potatoes	13.24 ± 1.39	11.05 ± 0.82	11.58 ± 0.46	12.63 ± 0.49	12.35 ± 0.65	0.5206
Sweet foods and sugars	4.72 ± 0.36	5.35 ± 0.41	6.29 ± 0.29	5.52 ± 0.22	5.79 ± 0.31	0.5509
Oil and fats	0.01 ± 0.01	0.02 ± 0.01	0.04 ± 0.01	0.05 ± 0.01	0.07 ± 0.02	0.002
Legumes	17.48 ± 1.01	17.94 ± 0.92	17.04 ± 0.44	19.64 ± 0.54	22.11 ± 0.82	<0.0001
Nuts	0.25 ± 0.06	0.31 ± 0.06	0.31 ± 0.04	0.41 ± 0.04	0.56 ± 0.08	<0.0001
Fruits	145.57 ± 11.27	131.30 ± 7.03	145.17 ± 5.20	176.94 ± 5.57	168.48 ± 7.75	<0.0001
Vegetables	41.86 ± 2.12	39.25 ± 2.21	45.75 ± 1.21	51.96 ± 1.24	51.58 ± 1.64	<0.0001
Mushrooms	3.45 ± 0.37	3.38 ± 0.57	4.12 ± 0.20	5.33 ± 0.23	5.78 ± 0.38	<0.0001
Meats	16.43 ± 1.02	18.71 ± 0.94	23.83 ± 0.81	26.09 ± 0.76	26.03 ± 0.96	<0.0001
Eggs	4.53 ± 0.50	4.93 ± 0.41	5.95 ± 0.24	7.72 ± 0.30	7.66 ± 0.46	<0.0001
Fish and shellfish	14.72 ± 1.42	13.69 ± 0.73	19.30 ± 0.57	24.08 ± 0.60	27.08 ± 0.98	<0.0001
Seaweed	0.35 ± 0.03	0.44 ± 0.02	0.74 ± 0.02	1.40 ± 0.03	2.16 ± 0.07	<0.0001
Dairy	55.06 ± 5.00	53.08 ± 3.77	69.63 ± 2.73	77.29 ± 2.87	69.51 ± 3.12	0.003
Beverages	27.24 ± 3.26	38.42 ± 4.67	40.01 ± 2.09	48.94 ± 2.50	46.73 ± 2.57	0.0001

Values were expressed as means ± standard error. Food intakes were expressed as g/1000 kcal. The *p* for trend was calculated by assigning the median laver intake (g/day) within each intake category and modeling this value as a continuous variable in the general linear model. Participants were classified into five laver-intake frequency categories: rarely (≤1 time/month), 2–3 times/month, 1–2 times/week, 3–6 times/week, and almost every day (1–2 times/day).

**Table 3:** Hazard ratios and 95% confidence intervals for incident abdominal obesity according to laver-intake frequency categories.

	Laver Intake Frequency Categories					
	Rarely (≤1 Time/Month)	2–3 Times/Month	1–2 Times/Week	3–6 Times/Week	Almost Every Day (1–2 Times/Day)	*p*-Trend
Men						
No. of cases	150 (43.48)	152 (39.28)	345 (38.00)	344 (39.95)	211 (38.57)	0.3288
Person-years	3551.0	4440.5	10,214.4	9920.7	5875.9	
Model 1	1 (reference)	0.81 (0.65–1.02)	0.80 (0.66–0.97)	1.11 (0.95–1.31)	1.16 (0.96–1.41)	0.9106
Model 2	1 (reference)	0.84 (0.67–1.05)	0.78 (0.64–0.95)	0.68 (0.56–0.83)	0.69 (0.56–0.86)	0.0045
Model 3	1 (reference)	0.86 (0.68–1.07)	0.80 (0.65–0.97)	0.71 (0.58–0.86)	0.72 (0.58–0.89)	0.0099
Women						
No. of cases	150 (55.76)	142 (52.01)	299 (44.76)	340 (44.44)	186 (42.86)	0.0002
Person-years	2600.2	2822.6	7175.6	8504.5	4665.1	
Model 1	1 (reference)	0.87 (0.69–1.10)	0.73 (0.60–0.88)	0.71 (0.58–0.86)	0.69 (0.56–0.86)	0.0056
Model 2	1 (reference)	0.91 (0.72–1.15)	0.81 (0.66–0.98)	0.75 (0.62–0.91)	0.67 (0.53–0.83)	0.0004
Model 3	1 (reference)	0.91 (0.72–1.15)	0.81 (0.67–0.99)	0.76 (0.62–0.93)	0.67 (0.54–0.84)	0.0005

Participants were classified into five laver-intake frequency categories. The lowest laver-intake frequency category was used as the reference group. The number of cases is presented as *n* (%). Model 1 was unadjusted. Model 2 was adjusted for age, BMI, alcohol consumption, smoking status, education level, physical activity level (MET-h/d), and total energy intake; menopausal status was additionally included in the model for women. Model 3 was adjusted for the covariates in Model 2 + HEI-2020 score. The *p* for trend was calculated by assigning the median laver intake (g/day) within each intake category and modeling this value as a continuous variable. BMI, body mass index; MET-h/d, metabolic equivalent task-hours per day; HEI-2020, healthy eating index-2020.

## Data Availability

Data described in the manuscript and the code book will be made available upon request pending approval by the Korea Centers for Disease Control.

## Abbreviations

The following abbreviations are used in this manuscript:

WC	Waist Circumference
KoGES	Korean Genome and Epidemiology Study
SQFFQ	Semi-Quantitative Food Frequency Questionnaire
KSSO	Korean Society for the Study of Obesity
MET	Metabolic Equivalent
SEs	Standard Errors
HEI	Healthy Eating Index
BMI	Body Mass Index
HRs	Hazard Ratios
CIs	Confidence Intervals
KRW	Korean Won
ELANS	Latin American Health and Nutrition Study
AMPK	AMP-activated protein kinase

## Appendix A

**Table A1:** Food item classification.

Food Groups	Food Items
Grains	Cooked rice well milled, cooked rice with soybean, cooked rice with other cereals, ramyon, wheat noodles, chajangmyon, buckwheat vermicelli/buckwheat noodles, dumpling, white rice cake/rice cake with soup, other rice cakes, loaf bread, bread with small red beans, other breads, pizza/hamburger, parched cereal powder, cereals, cakes/chocopie
Refined grains	Cooked rice well milled, ramyon, wheat noodles, chajangmyon, buckwheat vermicelli/buckwheat noodles, dumpling, white rice cake/rice cake with soup, other rice cakes, loaf bread, bread with small red beans, other breads, pizza/hamburger, parched cereal powder, cereals, cakes/chocopie
Whole grains	Cooked rice with soybean, cooked rice with other cereals
Potatoes	Potatoes, sweet potatoes, starch vermicelli
Sweet foods and sugars	Cookie/cracker/snack, candy/chocolate, coffee sugar
Oil and fats	Jam/honey/butter/margarine
Legumes	Beans/beans cooked in soy sauce, soup and stew with soybean paste/soybean paste, tofu, soybean milk
Nuts	Peanuts/almonds/pine nuts
Fruits	Strawberry, oriental melon/melon, watermelon, peach/plum, banana, persimmon/dried persimmon, tangerine, pear/pear juice, apple/apple juice, orange/orange juice, grape/grape juice, tomato/tomato juice
Vegetables	Korean cabbage kimchi, kkakduki/small radish kimchi, kimchi/radish with water, other kimchies (green onion/kodulbbagi/mustard leaves), green pepper, pepper leaves, spinach, lettuce, perilla leaf, crown daisy/water dropwort, other green vegetables, radish/salted radish, deoduck/doraji, onion,/Korean cabbages/Korean cabbage soup, cucumber, bean sprouts, carrot/carrot juice, pumpkin mature/pumpkin juice, pumpkin immature, vegetable juice, bracken/bracken stem
Salted vegetables	Korean cabbage kimchi, kkakduki/small radish kimchi, kimchi/Radish with water, Other kimchies, radish/salted radish,
Mushrooms	Oyster mushroom, other mushrooms
Meats	Pork belly, roasted pork, braised pork, processed meat(ham/sausage), edible viscera, steak/roasted beef, dog meat, fried chicken/chicken stew, beef soup with vegetables
Eggs	Eggs
Fish and shellfish	Sushi, mackerel/pacific saury/Spanish mackerel, hair tail, eel, yellow croaker, Alaska pollack, squid/octopus, dried anchovy, canned tuna, salt-fermented fish, clam/whelk, oyster, crab, shrimp, fish paste/crab flavored
Seaweeds	Dried laver, kelp/sea mustard
Dairy	Milk, yogurt, ice cream, cheese, coffee cream
Beverage	Carbonated drinks, coffee, soybean, green tea, other drinks

**Table A2:** Nutrient intake according to laver-intake frequency categories.

	Laver Intake Frequency Categories					
	Rarely (≤1 Time/Month)	2–3 Times/Month	1–2 Times/Week	3–6 Times/Week	Almost Every Day (1–2 Times/Day)	*p*-Trend
Men						
Total energy (kcal/day)	1790.65 ± 34.09	1784.14 ± 29.39	1977.11 ± 20.02	2073.76 ± 21.46	2175.32 ± 29.72	<0.0001
Protein (g/1000 kcal/day)	30.45 ± 0.28	30.90 ± 0.24	33.54 ± 0.17	35.34 ± 0.18	36.31 ± 0.23	<0.0001
Fat (g/1000 kcal/day)	13.88 ± 0.31	14.76 ± 0.27	17.11 ± 0.17	18.18 ± 0.18	18.51 ± 0.22	<0.0001
Carbohydrate (g/1000 kcal/day)	184.34 ± 0.88	181.98 ± 0.77	174.71 ± 0.50	171.20 ± 0.54	169.79 ± 0.67	<0.0001
Ca (mg/1000 kcal/day)	196.12 ± 4.49	197.09 ± 4.32	225.78 ± 2.55	248.63 ± 2.85	271.21 ± 3.75	<0.0001
*p* (mg/1000 kcal/day)	469.35 ± 4.21	470.81 ± 3.78	505.72 ± 2.38	532.27 ± 2.49	553.93 ± 3.27	<0.0001
Fe (mg/1000 kcal/day)	4.69 ± 0.07	4.74 ± 0.06	5.19 ± 0.04	5.68 ± 0.04	5.98 ± 0.05	<0.0001
K (mg/1000 kcal/day)	1127.17 ± 17.74	1118.36 ± 15.63	1203.10 ± 9.54	1307.26 ± 10.45	1373.20 ± 12.90	<0.0001
Na (mg/1000 kcal/day)	1711.15 ± 45.03	1520.16 ± 34.72	1644.55 ± 21.71	1727.52 ± 23.97	1830.75 ± 27.96	<0.0001
Vitamin A (R.E./1000 kcal/day)	224.82 ± 7.98	218.84 ± 6.91	251.22 ± 4.26	294.49 ± 5.29	333.16 ± 7.37	<0.0001
Vitamin B1 (mg/1000 kcal/day)	0.60 ± 0.01	0.59 ± 0.01	0.63 ± 0.00	0.66 ± 0.00	0.68 ± 0.01	<0.0001
Vitamin B2 (mg/1000 kcal/day)	0.43 ± 0.01	0.44 ± 0.01	0.49 ± 0.00	0.54 ± 0.00	0.57 ± 0.00	<0.0001
Niacin (mg/1000 kcal/day)	7.30 ± 0.08	7.36 ± 0.07	7.93 ± 0.05	8.37 ± 0.05	8.68 ± 0.06	<0.0001
Vitamin C (mg/1000 kcal/day)	53.24 ± 1.68	53.01 ± 1.59	54.37 ± 0.83	62.21 ± 1.04	64.85 ± 1.30	<0.0001
Zinc (mg/1000 kcal/day)	4.19 ± 0.06	4.18 ± 0.05	4.44 ± 0.03	4.67 ± 0.04	4.79 ± 0.05	<0.0001
Vitamin B6 (mg/1000 kcal/day)	0.85 ± 0.01	0.82 ± 0.01	0.88 ± 0.01	0.93 ± 0.01	0.95 ± 0.01	<0.0001
Folate (μg/1000 kcal/day)	105.87 ± 2.17	101.75 ± 1.75	114.48 ± 1.15	127.91 ± 1.37	141.09 ± 1.83	<0.0001
Retinol (μg/1000 kcal/day)	21.36 ± 1.02	26.30 ± 1.06	33.91 ± 0.70	38.27 ± 0.90	38.46 ± 0.98	<0.0001
Carotene (μg/1000 kcal/day)	1183.84 ± 49.85	1125.23 ± 42.17	1266.10 ± 26.32	1505.96 ± 32.72	1750.72 ± 47.46	<0.0001
Fiber (g/1000 kcal/day)	3.35 ± 0.07	3.20 ± 0.06	3.30 ± 0.03	3.53 ± 0.04	3.62 ± 0.05	<0.0001
Vitamin E (mg/1000 kcal/day)	4.07 ± 0.08	4.13 ± 0.06	4.47 ± 0.04	4.88 ± 0.05	5.05 ± 0.06	<0.0001
Cholesterol (mg/1000 kcal/day)	58.12 ± 2.21	65.12 ± 2.03	86.25 ± 1.45	101.39 ± 1.75	107.91 ± 2.20	<0.0001
Taurine (mg/1000 kcal/day)	43.15 ± 2.68	47.70 ± 2.49	63.99 ± 1.79	81.90 ± 2.10	91.67 ± 2.92	<0.0001
Women						
Total energy (kcal/day)	1620.65 ± 36.11	1623.17 ± 35.53	1765.72 ± 21.04	1870.01 ± 22.27	2034.12 ± 30.35	<0.0001
Protein (g/1000 kcal/day)	30.57 ± 0.38	30.92 ± 0.30	33.00 ± 0.20	34.94 ± 0.20	36.16 ± 0.28	<0.0001
Fat (g/1000 kcal/day)	12.56 ± 0.34	13.39 ± 0.32	15.43 ± 0.22	16.59 ± 0.21	16.86 ± 0.27	<0.0001
Carbohydrate (g/1000 kcal/day)	187.78 ± 0.98	185.43 ± 0.90	179.53 ± 0.62	175.82 ± 0.62	174.07 ± 0.80	<0.0001
Ca (mg/1000 kcal/day)	220.93 ± 7.49	214.55 ± 5.55	245.14 ± 3.55	273.68 ± 3.62	285.73 ± 4.87	<0.0001
*p* (mg/1000 kcal/day)	499.43 ± 6.49	497.18 ± 5.18	525.15 ± 3.11	554.38 ± 3.13	569.73 ± 4.45	<0.0001
Fe (mg/1000 kcal/day)	5.08 ± 0.10	5.10 ± 0.09	5.43 ± 0.05	5.92 ± 0.05	6.29 ± 0.07	<0.0001
K (mg/1000 kcal/day)	1217.64 ± 28.77	1186.50 ± 21.62	1287.60 ± 12.82	1413.19 ± 12.61	1426.76 ± 17.80	<0.0001
Na (mg/1000 kcal/day)	1581.21 ± 54.42	1483.70 ± 43.17	1572.43 ± 26.39	1654.12 ± 23.20	1748.96 ± 33.18	<0.0001
Vitamin A (R.E./1000 kcal/day)	217.72 ± 10.50	202.67 ± 7.55	240.51 ± 5.40	294.64 ± 5.90	319.86 ± 7.70	<0.0001
Vitamin B1 (mg/1000 kcal/day)	0.58 ± 0.01	0.58 ± 0.01	0.61 ± 0.00	0.65 ± 0.00	0.66 ± 0.01	<0.0001
Vitamin B2 (mg/1000 kcal/day)	0.44 ± 0.01	0.44 ± 0.01	0.50 ± 0.00	0.56 ± 0.01	0.58 ± 0.01	<0.0001
Niacin (mg/1000 kcal/day)	7.21 ± 0.10	7.31 ± 0.09	7.74 ± 0.06	8.22 ± 0.05	8.47 ± 0.08	<0.0001
Vitamin C (mg/1000 kcal/day)	61.70 ± 2.83	58.15 ± 2.01	63.58 ± 1.30	73.51 ± 1.40	72.41 ± 1.89	<0.0001
Zinc (mg/1000 kcal/day)	4.11 ± 0.06	4.17 ± 0.05	4.36 ± 0.03	4.52 ± 0.03	4.62 ± 0.06	<0.0001
Vitamin B6 (mg/1000 kcal/day)	0.88 ± 0.02	0.86 ± 0.01	0.91 ± 0.01	0.96 ± 0.01	0.98 ± 0.01	<0.0001
Folate (μg/1000 kcal/day)	114.73 ± 3.27	111.82 ± 2.32	120.77 ± 1.51	136.63 ± 1.47	149.64 ± 2.16	<0.0001
Retinol (μg/1000 kcal/day)	27.51 ± 1.68	27.68 ± 1.37	34.76 ± 0.88	41.56 ± 1.01	39.52 ± 1.48	<0.0001
Carotene (μg/1000 kcal/day)	1108.11 ± 61.71	1008.61 ± 43.21	1205.05 ± 33.66	1491.31 ± 37.61	1640.38 ± 45.44	<0.0001
Fiber (g/1000 kcal/day)	3.66 ± 0.09	3.52 ± 0.07	3.61 ± 0.04	3.82 ± 0.04	3.89 ± 0.06	<0.0001
Vitamin E (mg/1000 kcal/day)	4.20 ± 0.11	4.23 ± 0.10	4.55 ± 0.05	5.06 ± 0.05	5.23 ± 0.07	<0.0001
Cholesterol (mg/1000 kcal/day)	62.84 ± 3.42	64.99 ± 2.78	82.71 ± 1.74	99.90 ± 1.89	104.90 ± 2.75	<0.0001
Taurine (mg/1000 kcal/day)	54.98 ± 5.44	49.44 ± 3.06	68.14 ± 2.32	87.07 ± 2.40	99.55 ± 3.65	<0.0001

Values are expressed as the mean ± standard error. Nutrient intakes are expressed per 1000 kcal. The *p* for trend was calculated by assigning the median laver intake (g/day) within each intake category and modeling this value as a continuous variable in the general linear model. Participants were classified into five laver-intake frequency categories: rarely (≤1 time/month), 2–3 times/month, 1–2 times/week, 3–6 times/week, and almost every day (1–2 times/day).

## References

[1] Alfaris N., Alqahtani A.M., Alamuddin N., Rigas G. (2023). Global Impact of Obesity. Gastroenterol. Clin. N. Am..

[2] Prospective Studies Collaboration (2009). Body-mass index and cause-specific mortality in 900,000 adults: Collaborative analyses of 57 prospective studies. Lancet.

[3] Ibrahim M.M. (2010). Subcutaneous and visceral adipose tissue: Structural and functional differences. Obes. Rev..

[4] Fontana L., Eagon J.C., Trujillo M.E., Scherer P.E., Klein S. (2007). Visceral fat adipokine secretion is associated with systemic inflammation in obese humans. Diabetes.

[5] Després J.-P., Lemieux I. (2006). Abdominal obesity and metabolic syndrome. Nature.

[6] Clinton S.K., Giovannucci E.L., Hursting S.D. (2020). The World Cancer Research Fund/American Institute for Cancer Research third expert report on diet, nutrition, physical activity, and cancer: Impact and future directions. J. Nutr..

[7] Lee K.W., Kang M.-S., Lee S.J., Kim H.-R., Jang K.-A., Shin D. (2023). Prospective associations between dietary patterns and abdominal obesity in middle-aged and older Korean adults. Foods.

[8] Jensen M.G., Kristensen M., Astrup A. (2012). Effect of alginate supplementation on weight loss in obese subjects completing a 12-wk energy-restricted diet: A randomized controlled trial. Am. J. Clin. Nutr..

[9] Magwaza S.t.N., Islam M.S. (2025). Mechanisms behind the anti-diabetic and anti-obesity effects of seaweeds or macroalgae and their bioactive compounds. World J. Diabetes.

[10] Holdt S.L., Kraan S. (2011). Bioactive compounds in seaweed: Functional food applications and legislation. J. Appl. Phycol..

[11] Peñalver R., Lorenzo J.M., Ros G., Amarowicz R., Pateiro M., Nieto G. (2020). Seaweeds as a functional ingredient for a healthy diet. Mar. Drugs.

[12] Chen L., Liu R., He X., Pei S., Li D. (2021). Effects of brown seaweed polyphenols, a class of phlorotannins, on metabolic disorders via regulation of fat function. Food Funct..

[13] Lange K.W., Hauser J., Nakamura Y., Kanaya S. (2015). Dietary seaweeds and obesity. Food Sci. Hum. Wellness.

[14] Hwang E.K., Park C.S. (2020). Seaweed cultivation and utilization of Korea. Algae.

[15] FAO (2018). The Global Status of Seaweed Production, Trade and Utilization.

[16] Park H., Lee K.W., Shin D. (2022). Association of seaweed consumption with metabolic syndrome and its components: Findings from the Korean Genome and Epidemiology Study. Foods.

[17] Park J.-K., Woo H.W., Kim M.K., Shin J., Lee Y.-H., Shin D.H., Shin M.-H., Choi B.Y. (2021). Dietary iodine, seaweed consumption, and incidence risk of metabolic syndrome among postmenopausal women: A prospective analysis of the Korean Multi-Rural Communities Cohort Study (MRCohort). Eur. J. Nutr..

[18] Khaliq M., Noor Z., Moazzam M., Saeed F., Tul-Zohra K., Tariq E., Muhammad Shahbaz H., Khaliq K. (2026). Navigating the potential of algal peptides: Health effects, market applications, and scientific challenges. Ann. Med..

[19] Admassu H., Gasmalla M.A.A., Yang R., Zhao W. (2018). Bioactive peptides derived from seaweed protein and their health benefits: Antihypertensive, antioxidant, and antidiabetic properties. J. Food Sci..

[20] Shams-White M.M., Pannucci T.E., Lerman J.L., Herrick K.A., Zimmer M., Mathieu K.M., Stoody E.E., Reedy J. (2023). Healthy Eating Index-2020: Review and update process to reflect the Dietary Guidelines for Americans, 2020–2025. J. Acad. Nutr. Diet..

[21] Tande D.L., Magel R., Strand B.N. (2010). Healthy Eating Index and abdominal obesity. Public Health Nutr..

[22] Kim Y., Han B.G., KoGES Group (2017). Cohort profile: The Korean Genome and Epidemiology Study (KoGES) Consortium. Int. J. Epidemiol..

[23] Seo M.H., Lee W.-Y., Kim S.S., Kang J.-H., Kang J.-H., Kim K.K., Kim B.-Y., Kim Y.-H., Kim W.-J., Kim E.M. (2019). 2018 Korean Society for the Study of Obesity guideline for the management of obesity in Korea. J. Obes. Metab. Syndr..

[24] Ahn Y., Kwon E., Shim J., Park M., Joo Y., Kimm K., Park C., Kim D. (2007). Validation and reproducibility of food frequency questionnaire for Korean Genome Epidemiologic Study. Eur. J. Clin. Nutr..

[25] Shim Y.J., Paik H.Y. (2009). Reanalysis of 2007 Korean National Health and Nutrition Examination Survey (2007 KNHANES) results by CAN-Pro 3.0 nutrient database. Korean J. Nutr..

[26] National Institute of Agricultural Sciences (2021). Food Composition Table.

[27] Krebs-Smith S.M., Pannucci T.E., Subar A.F., Kirkpatrick S.I., Lerman J.L., Tooze J.A., Wilson M.M., Reedy J. (2018). Update of the Healthy Eating Index: HEI-2015. J. Acad. Nutr. Diet..

[28] Zang L., Baharlooeian M., Terasawa M., Shimada Y., Nishimura N. (2023). Beneficial effects of seaweed-derived components on metabolic syndrome via gut microbiota modulation. Front. Nutr..

[29] Gomez G., Kovalskys I., Leme A.C.B., Quesada D., Rigotti A., Cortes Sanabria L.Y., Yepez Garcia M.C., Liria-Domínguez M.R., Herrera-Cuenca M., Fisberg R.M. (2021). Socioeconomic status impact on diet quality and body mass index in eight Latin American countries: ELANS study results. Nutrients.

[30] Fujii H., Iwase M., Ohkuma T., Ogata-Kaizu S., Ide H., Kikuchi Y., Idewaki Y., Joudai T., Hirakawa Y., Uchida K. (2013). Impact of dietary fiber intake on glycemic control, cardiovascular risk factors and chronic kidney disease in Japanese patients with type 2 diabetes mellitus: The Fukuoka Diabetes Registry. Nutr. J..

[31] Sawicki C.M., Jacques P.F., Lichtenstein A.H., Rogers G.T., Ma J., Saltzman E., McKeown N.M. (2021). Whole- and refined-grain consumption and longitudinal changes in cardiometabolic risk factors in the Framingham Offspring Cohort. J. Nutr..

[32] Radhika G., Van Dam R.M., Sudha V., Ganesan A., Mohan V. (2009). Refined grain consumption and the metabolic syndrome in urban Asian Indians (Chennai Urban Rural Epidemiology Study 57). Metabolism.

[33] Parikh S., Pollock N.K., Bhagatwala J., Guo D.-H., Gutin B., Zhu H., Dong Y. (2012). Adolescent fiber consumption is associated with visceral fat and inflammatory markers. J. Clin. Endocrinol. Metab..

[34] Slavin J.L. (2005). Dietary fiber and body weight. Nutrition.

[35] Slavin J., Green H. (2007). Dietary fibre and satiety. Nutr. Bull..

[36] Baer D.J., Rumpler W.V., Miles C.W., Fahey G.C. (1997). Dietary fiber decreases the metabolizable energy content and nutrient digestibility of mixed diets fed to humans. J. Nutr..

[37] Weickert M.O., Pfeiffer A.F. (2018). Impact of dietary fiber consumption on insulin resistance and the prevention of type 2 diabetes. J. Nutr..

[38] Den Besten G., Van Eunen K., Groen A.K., Venema K., Reijngoud D.-J., Bakker B.M. (2013). The role of short-chain fatty acids in the interplay between diet, gut microbiota, and host energy metabolism. J. Lipid Res..

[39] Cho T.J., Rhee M.S. (2020). Health functionality and quality control of laver (Porphyra, Pyropia): Current issues and future perspectives as an edible seaweed. Mar. Drugs.

[40] Wang X., Dong J., Liang W., Fang Y., Liang M., Xu L., Sun W., Li X. (2022). Porphyran from Porphyra haitanensis alleviates obesity by reducing lipid accumulation and modulating gut microbiota homeostasis. Front. Pharmacol..

[41] Cao J., Wang S., Yao C., Xu Z., Xu X. (2016). Hypolipidemic effect of porphyran extracted from Pyropia yezoensis in ICR mice with high fatty diet. J. Appl. Phycol..

[42] Choi S.-Y., Lee S.Y., Jang D.H., Lee S.J., Cho J.-Y., Kim S.-H. (2020). Inhibitory effects of Porphyra dentata extract on 3T3-L1 adipocyte differentiation. J. Anim. Sci. Technol..

[43] Tsuge K., Okabe M., Yoshimura T., Sumi T., Tachibana H., Yamada K. (2004). Dietary effects of porphyran from Porphyra yezoensis on growth and lipid metabolism of Sprague-Dawley rats. Food Sci. Technol. Res..

[44] Jung B.-M., Jung K.-J. (2005). Effect of porphyran drink on serum and liver cholesterol contents in hypercholesterolemic rat. J. Korean Soc. Food Sci. Nutr..

[45] Lee J.-S., Lee M.-H., Koo J.-G. (2010). Effects of porphyran and insoluble dietary fiber isolated from laver, Porphyra yezoensis, on lipid metabolism in rats fed high fat diet. Korean J. Food Nutr..

[46] Guo Y.-Y., Li B.-Y., Peng W.-Q., Guo L., Tang Q.-Q. (2019). Taurine-mediated browning of white adipose tissue is involved in its anti-obesity effect in mice. J. Biol. Chem..

[47] Sun Q., Wang J., Wang H., Yu H., Wan K., Ma F., Wang R. (2024). Effect of long-term taurine supplementation on the lipid and glycaemic profile in adults with overweight or obesity: A systematic review and meta-analysis. Nutrients.

[48] Karikkakkavil Prakashan A.D., Muthukumar S.P., Martin A. (2024). Taurine activates SIRT1/AMPK/FOXO1 signaling pathways to favorably regulate lipid metabolism in C57BL6 obese mice. Mol. Nutr. Food Res..

[49] Tsuboyama-Kasaoka N., Shozawa C., Sano K., Kamei Y., Kasaoka S., Hosokawa Y., Ezaki O. (2006). Taurine (2-aminoethanesulfonic acid) deficiency creates a vicious circle promoting obesity. Endocrinology.

[50] Reis J.P., Macera C.A., Araneta M.R., Lindsay S.P., Marshall S.J., Wingard D.L. (2009). Comparison of overall obesity and body fat distribution in predicting risk of mortality. Obesity.

[51] Lee G., Choi S., Park S.M. (2021). Association of waist circumference with muscle and fat mass in adults with a normal body mass index. Nutr. Res. Pract..

